# Semi-empirical parameterization of HI/p L-shell X-ray production cross section ratios in Bi for Heavy Ion PIXE

**DOI:** 10.1038/s41598-023-48217-y

**Published:** 2023-11-28

**Authors:** M. C. Masekane, M. Msimanga, I. Bogdanović Radović, M. Madhuku, S. J. Moloi

**Affiliations:** 1 Department of Physics, University of South Africa, P Bag X90, Florida, 1710 South Africa; 2 Department of Physics, University of Zagreb, Bijenićka C. 32, 10000 Zagreb, Croatia; 3 Ruđer Bošković Institute, P.O. Box 1016, 10000 Zagreb, Croatia; 4 iThemba LABS TAMS, National Research Foundation, P Bag 11, WITS, Johannesburg, 2050 South Africa; 5 Physics Department, Tshwane University of Technology, P Bag X680, Pretoria, 0001 South Africa

**Keywords:** Atomic and molecular physics, Techniques and instrumentation, Characterization and analytical techniques

## Abstract

Quantitative analysis of materials from Heavy Ion PIXE spectra remains impeded by the lack of reliable X-ray production cross section (XPCS) data. Although efforts at experimental Heavy Ion induced XPCS measurements still continue, Multiple Ionisation (MI) effects, which are not fully described by theory, render simulations of heavy ion PIXE data unreliable for large Z_1_/Z_2_ collisions, especially at low energies. This is also exacerbated by the random selection of projectile-target combinations for measured and reported experimental data available to validate theory. This study explored heavy ion induced X-ray production cross section deviations from those induced by protons at the same ion velocity. This enabled evaluations of the degree to which cross sections are enhanced through MI effects, with the aim of predicting XPCS due to heavy ion impact. The evaluation was carried out through the scaling of experimental heavy ion to theoretical proton cross section ratios (*R*), which were then used for the interpolation of XPCS in the same target element for ‘missing’ projectiles within the range of evaluation. Here we present measurements of heavy ion induced total L-shell XPCS in Bi, carried out to determine HI/p MI induced deviations due to C, F, Cl and Ti projectiles at an ion velocity range of (0.2–1.0) MeV/nucleon.

## Introduction

The Total Ion Beam Analysis (TIBA) approach to analyses of thin film materials, where both particle scattering and photon emission processes are combined to provide a consolidated description of a material is bound to greatly enhance the efficacy of nuclear analytical techniques^1,2^. Heavy Ion TIBA in particular, has the potential to improve the degree to which atomic and structural information is extracted from a material matrix^3^. One potential heavy ion TIBA configuration is that which may be inclusive of Heavy Ion Particle Induced X-ray Emission (HI-PIXE) spectroscopy, where PIXE is performed concurrently with heavy ion based IBA techniques that provide thin layer (nm range) structural and/or surface molecular data (e.g. Elastic Recoil Detection Analysis (ERDA) or Secondary Ion Mass Spectrometry (SIMS))^1,3,4^. PIXE is routinely carried out with techniques such as Rutherford Backscattering (RBS) or Particle Induced γ-Ray emission (PIGE) spectroscopy in many IBA laboratories, but using light projectiles (such as protons or helium ions) instead. The potential of heavy ion (~ Z ≥ 6) probes to boost IBA technique capabilities lies largely on the relatively higher ion-atom interaction cross sections compared to those due to light projectiles^5,6^. This especially implies higher sensitivity for the detection of both light and high Z_2_ elements emanating from the same projectile-target interactions, e.g. recoil production. Due to Multiple Ionisation effects, enhanced X-ray production cross sections at the same projectile velocity as protons mean that PIXE using heavy ions may in some cases yield comparable X-ray fluorescence for low velocity heavy ions below 1 MeV/nucleon as with faster protons^7–9^. This is because, fundamentally, protonic ionisation, largely due to the deflection of the proton nuclear charge about the target nuclear mean field, is not the same as that which is due to heavier ions. Slow moving heavy ions, which have larger nuclear and electron potentials, can be characterized as having longer dwell times within the vicinity of the target nuclear mean field^10^. This makes the target atom prone to several ionisations in a single collision event, which goes beyond Direct Ionisation and may include other ionisation modes such as electron capture^11^.

The widespread implementation of PIXE spectroscopy has been vastly reported on, and largely relies on the existence of reliable theoretical predictions of X-ray production cross section (XPCS) data at usual PIXE energies ((1.0–3.0) MeV). These theoretical formulations include widely adopted frameworks such as the ECPSSR theory by Brandt and Lapicki, which is based on the Plane Wave Born Approximation (PWBA)^12–15^. The ECPSSR model essentially extends the first order Born approximation by including corrections for Energy loss (E) as the projectile traverses the Coulomb potential (C) region of the target electron cloud, causing a Perturbation in the Stationary State (PSS) of the target atom. The model also treats the Relativistic nature (R) of the inner-shell electrons, and uses hydrogenic wave functions including the binding polarization effect^16,17^. The asymmetric collision between electron stripped light projectile and a heavier target atom is adequately described by Direct Ionisation (DI) theories such as the ECPSSR. With heavy ion-atom collisions however, intra-shell coupling effects which modify vacancy distributions for ionized target atoms due to heavy incident ions see higher discrepancies between experiment and DI theories based on protonic collisions. This is particularly the case at low energies, when the incident ion is only partially ionised^18–22^. These low energy discrepancies have been widely reported, including and showing evidence of pronounced Multiple Ionization (MI) effects in different heavy ion-atom collisions^18–23^.

The observed large experiment/theory X-ray production cross section ratios at low projectile velocities as a function of increasing projectile mass for various collision systems confirm the need for additional XPCS measurements^19,20^. Even so, explicit reliance on a sparsely populated experimental database largely scattered in published literature makes it somewhat impractical for analysis of experimental heavy ion PIXE spectra where theory is largely inadequate^21^. Therefore, continuous XPCS measurements alone in an effort at enriching the cross section database, are not enough. This is also exacerbated by the fact that it is a practically infeasible feat to try and cover all possible projectile-target combinations at all energies. While continuous cross section measurements are useful in sustaining efforts at enhancing the understanding of physical phenomena such as MI, an alternative approach to the use of theoretical models for approximating XPCS is required and this work presents an exploratory effort in that direction. This entails the development of semi-empirical approximations of XPCS based on the scaling of experimental heavy ion induced X-ray production cross section data and ECPSSR theoretical proton cross sections. These are also useful for assessing the degree to which ionisation is enhanced by heavy ion impact.

Presented in this paper are measurements of HI-XPCS induced by C, F, Cl, Ti in Bi. These are compared to data in the literature along with theoretical ECPSSR approximations. The measured cross section dataset is then used to interpolate XPCS within a defined projectile mass range, by using a semi-empirical approach that evaluates heavy ion to proton cross section ratios (HI/p) in matter, particularly for low velocity heavy ion–atom collisions.

## Approach

The normalized discrepancy between proton and heavy ion induced XPCS was used to quantify the projectile mass dependence on the collisions prompting characteristic X-ray emissions. This was carried out through systematic analyses of (experimental) heavy ion-to-(theoretical) proton (HI-p) XPCS ratio deviations *R*, defined by Eq. (1).

1 $$R=\frac{{\sigma }_{X(HI)}}{{\sigma }_{X(p)}}$$

*where σ*_*X(HI)*_* and σ*_*X(p)*_* are the heavy ion and proton induced XPCS respectively.*

**Figure 1:**
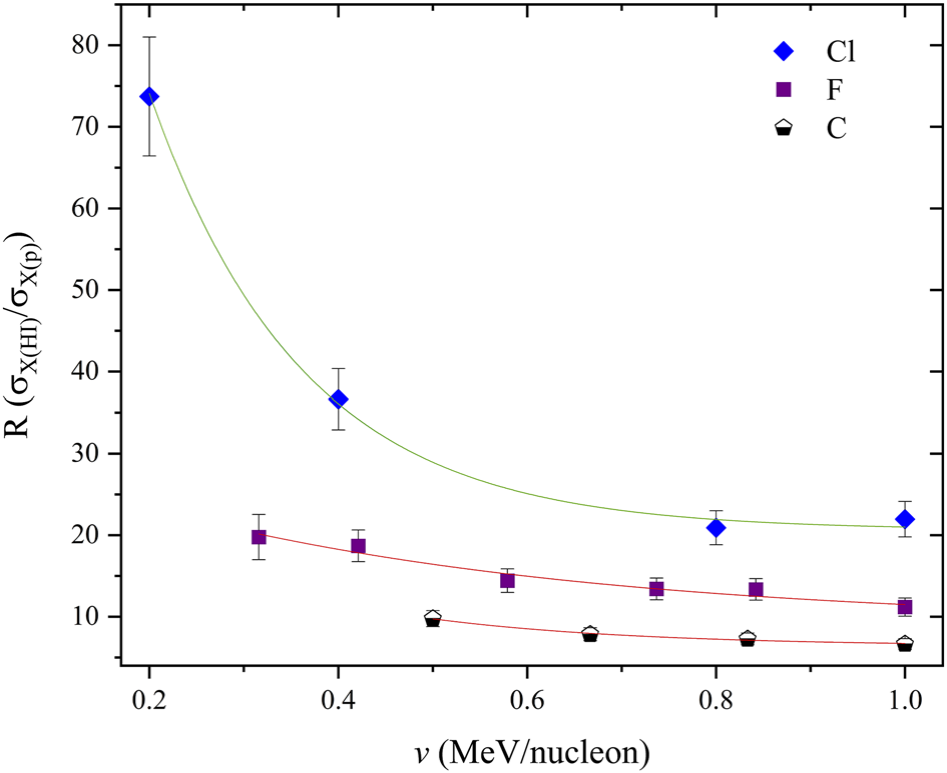
C, F and Cl R-values in Bi. Full lines are fits of the experimental values (symbols) using exponential decay functions.

The deviation of heavy ion from proton induced XPCS (seen in Fig. 1), shows strong screening effects, subsequently leading to pronounced Multiple Ionisation contributions for heavy ion–atom interactions, especially seen in large effect at low ion velocities. Higher electronic screening due to increasing ion mass, which as a result contributes to more ionisations at lower velocities compared to protons, can clearly be seen in Fig. 1.

The reduction in these deviations (*characterized by an exponential decay function of R-values*) as the ion velocity increases is attributed to increasing projectile charge states (i.e. used to obtain higher energies from the accelerator), where the reduced projectile electronic Coulomb potential leads to exponentially lower ion screening. Since these *R* deviations are sensitive to projectile screening, approximations may be well suited at lower energies where theoretical models have been seen to show the largest discrepancies.

The exponential carbon/proton trend, which resembles a linear-like trend compared to the chlorine trend shown in Fig. 1, to some extent characterizes quasi-binary interactions, especially at higher ion energies where the *R* function becomes even more saturated. This is because carbon is much lighter than chlorine, such that collisions with a large target system like Bi remain asymmetric, with a comparatively lower degree of electronic screening. This extent of screening is low enough such that the treatment of the ion potential as a plane wave, along with energy loss and target perturbation corrections are largely valid using ECPSSR predictions. This assertion is drawn from experimental work reported in^5,6^, particularly where projectile charge states are above 3. Electronic perturbation is however high in the low energy limit due to higher screening, such that corrections by the ECPSSR become insufficient for some collision systems, even asymmetric ones. As this is the case, a similar exponential decay function for carbon (as with chlorine) is shown in Fig. 4 later in the text. The linear-like trend seen in Fig. 1 is therefore only but comparative, highlighting the degree to which screening is pronounced for heavier ion masses.

In the case of chlorine projectiles, large *R*-ratios at low projectile velocities point to a quasi-molecular interaction (i.e. overlapping of ion and target atom electron orbitals in the collision moment), which has been described by^23,24^. By considering the Cl–Bi collision system at low velocities it is possible to see how the extent of projectile ionization affects the* R*-ratio in that energy region. By the same token, when looking at the higher velocity region, the *R* trend becomes linear and clearly characteristic of the mass of the projectile. Therefore, comparative analyses through multi-parameter fittings of *R*-ratios can be used to approximate other mass dependent *R*-ratios for projectiles within a defined ion velocity range. The calculation of these R ratios, coupled with theoretical ECPSSR proton XPCS may then enable the interpolation of heavy ion induced X-ray production cross sections for the same target atom. This XPCS calculation approach was tested using C and F projectile ions in Bi to predict N, O, Na and Si induced XPCS in Bi over an ion velocity range of (0.2–1.0) MeV/nucleon.

## Experimental setup

**Figure 2:**
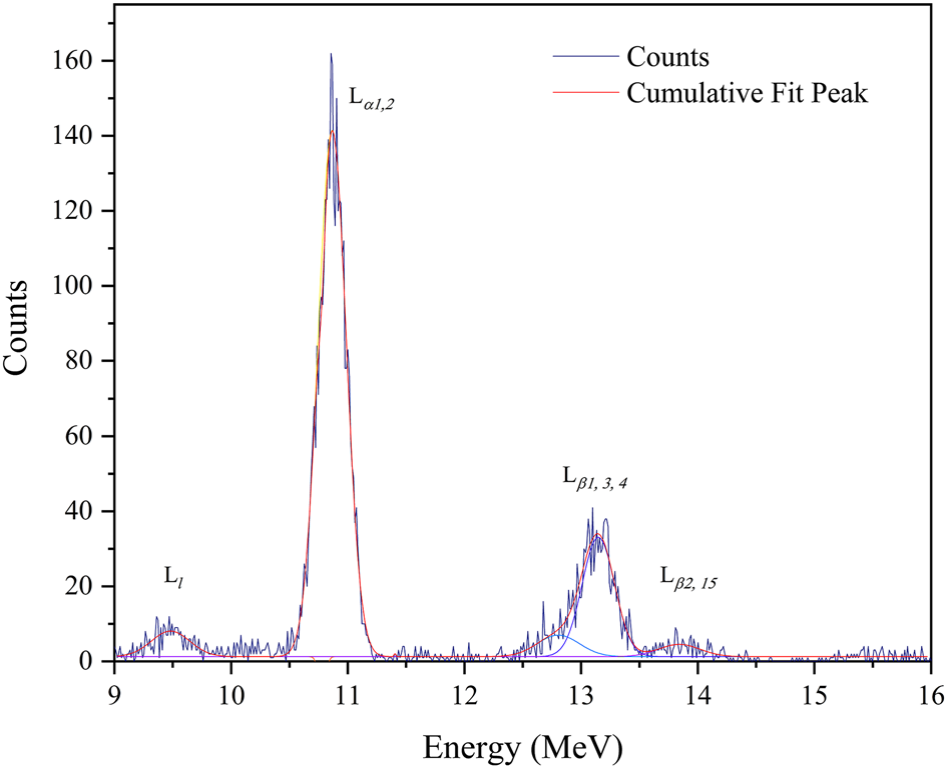
Bi L-shell X-ray spectra due to 0.67 MeV/nucleon ^48^Ti^6+^.

Experimental HI-XPCS measurements were carried out on the scanning nuclear microprobe of the 6MV Van de Graaf Tandem particle accelerator at the iThemba Laboratory for Accelerator Based Sciences (LABS). The material samples used were electron beam deposited ^209^Bi thin film layers (~ 185 nm) on 10 × 10 mm^2^ Mylar (Polyethylene Terephthalate) substrates. The film and substrate thicknesses (~ 20 μm) were selected to prevent X-ray emission from the non-hollow steel sample stage as well as from the target chamber walls. ^12^C^q+^ (q = 2, 3), ^19^F^q+^ (q = 2, 3, 4), ^35^Cl^q+^ (q = 2, 3, 4, 5) and ^48^Ti^q+^ (q = 5, 6) heavy ions from a SNICS ion source were accelerated to velocities ranging between (0.2–1.0) MeV/nucleon, with maximum currents of 1 nA obtained in the target chamber from a 2.0 mm^2^ broad beam. The beam spot size was broadened to lower heavy ion current densities such that radiation damage due to ion stopping in the sample was minimized. The accumulated charge was normalised to the ion backscattering intensities for all the projectiles used. The characteristic X-ray photons were counted using a Si(Li) detector (nominal resolution of 135 eV FWHM at 5.9 keV (Mn K_α_)) positioned at 135° relative to the 0° incident beam direction, while the backscattered particle yield was measured using a Canberra PIPS detector positioned at 150°. Integration of the obtained Gaussian distributions was carried out in Origin^®^ using the least square method for the extraction of X-ray yields, as illustrated in Fig. 2.

### X-ray production cross section evaluation

Heavy ion induced XPCS were evaluated through the analysis of PIXE and RBS data for individual projectile–target combinations through Eq. (2)^5^. A more comprehensive description of the calculation procedure is detailed in^24,25^.

2 $${\sigma }_{X}^{L}=\frac{{Y}_{X}}{{Y}_{B}} \cdot \frac{4\pi }{{\varepsilon }_{X}} \cdot \frac{{\Delta\Omega }_{B}}{{\Delta\Omega }_{X}} \cdot {F}_{HI}\left(\Delta E\right) \cdot {\sigma }_{B}({E}_{i})$$

Y_X_, Y_B_, ε_X_, Ω_B_, Ω_X_, F_HI_(ΔE) and σ_B_ are, respectively, the X-ray yield, incident ion backscattering yield, X-ray detection efficiency, RBS detector solid angle, X-ray detector solid angle, energy loss correction factor and the Rutherford cross section of the ion-atom collision^26,27^.

Absolute detector efficiencies ${\varepsilon }_{x}$ were determined from X-ray and backscattering yields induced by 2 MeV proton beams in different 100 nm thick mono-elemental targets (Sn, V, Gd and Bi backed by a Mylar substrate) (Eq. 3)^25^.

3 $${\varepsilon }_{x}=\frac{{Y}_{X(p)}}{{Y}_{B(p)}}\times \frac{{\sigma }_{B(p)}}{{\sigma }_{X(p)}}\times \frac{A}{{N}_{A}}\times \frac{{\Delta\Omega }_{B}}{{\mu }_{t}}\times \frac{{\rho }_{at}}{{\rho }_{m}}$$

Here the various symbols used are: ${\sigma }_{X(p)}$—ECPSSR theoretical X-ray production cross section; $A$—target atomic mass; ${N}_{A}$—Avogadro constant; ${\mu }_{t}$—coefficient of X-ray mass attenuation in the target layer^28^; ${\rho }_{at}$—atomic density^29^; and ${\rho }_{m}$—target mass density^29^.

Deviations in the measured heavy ion cross sections due to projectile energy loss through the Bi film were corrected for using Eq. (4)^30^.

4 $${F}_{HI}\left(\Delta E\right)=\frac{1}{\Delta x \cdot {\sigma }_{i}}\times {\int }_{{E}_{f}}^{{E}_{i}}\frac{{\sigma }_{i}(E)}{S(E)}dE$$

The integral in (4) was expanded using Simpson´s rule as detailed by Zuchiatti et al.^30^, where Δx, σi and S(E) are the target thickness, ionisation cross section and the total stopping power, respectively. Evaluation of measurement uncertainty included parameters used in Eq. (2), such as both the X-ray and backscattering yield, statistical uncertainties were below 3% for all projectiles after deadtime correction. The ion beam current was optimized to limit deadtime to 1%, which was corrected for by the data acquisition system. Additional considerations include measured detector efficiencies and solid angles, mathematically described in Eq. (5).

5 $$U=\sqrt{{\left(\frac{\delta {Y}_{X}}{{Y}_{X}}\right)}^{2}+{\left(\frac{{\delta\upsigma }_{X}}{{\upsigma }_{X}}\right)}^{2}+{\left(\frac{\delta {\varepsilon }_{X}}{{\varepsilon }_{X}}\right)}^{2}+{\left(\frac{\delta {Y}_{B}}{{Y}_{B}}\right)}^{2}+{\left(\frac{{\delta\upsigma }_{B}}{{\upsigma }_{B}}\right)}^{2}+{\left(\frac{{\delta\Omega }_{B}}{{\Omega }_{B}}\right)}^{2}}$$

The corrected experimental Total X-ray production cross sections are given in Table 1.

**Table 1:** Experimental X-ray production cross sections (barns) in Bi.

Z_1_ → Z_1_/Z_2_	v (MeV/nucleon)	Experimental L_Total_	ECPSSR (DI)
C → 0.057	0.50	2.8 ± 0.3	6.3
	0.67	7.3 ± 0.7	18.7
	0.83	15.2 ± 1.5	40.1
	1.00	25.3 ± 2.5	71.5
F → 0.091	0.32	0.47 ± 0.07	1.04
	0.42	2.3 ± 0.2	3.8
	0.58	7.7 ± 0.8	14.6
	0.68	15.1 ± 1.5	26.6
	0.74	17.7 ± 1.8	36.1
	0.84	28.8 ± 2.8	55.9
	1.00	42.5 ± 4.2	99.3
Cl → 0.17	0.20	0.045 ± 0.004	0.027
	0.60	3.3 ± 0.3	14
	0.80	37.9 ± 3.8	43.9
	1.00	83.5 ± 8.3	99
Ti → 0.23	0.31	1.34 ± 0.1	0.376
	0.42	5.26 ± 0.5	2.01
	0.50	14.3 ± 1.4	4.67
	0.58	29.6 ± 2.9	9.09
	0.67	51.0 ± 5.1	16.7

**Figure 3:**
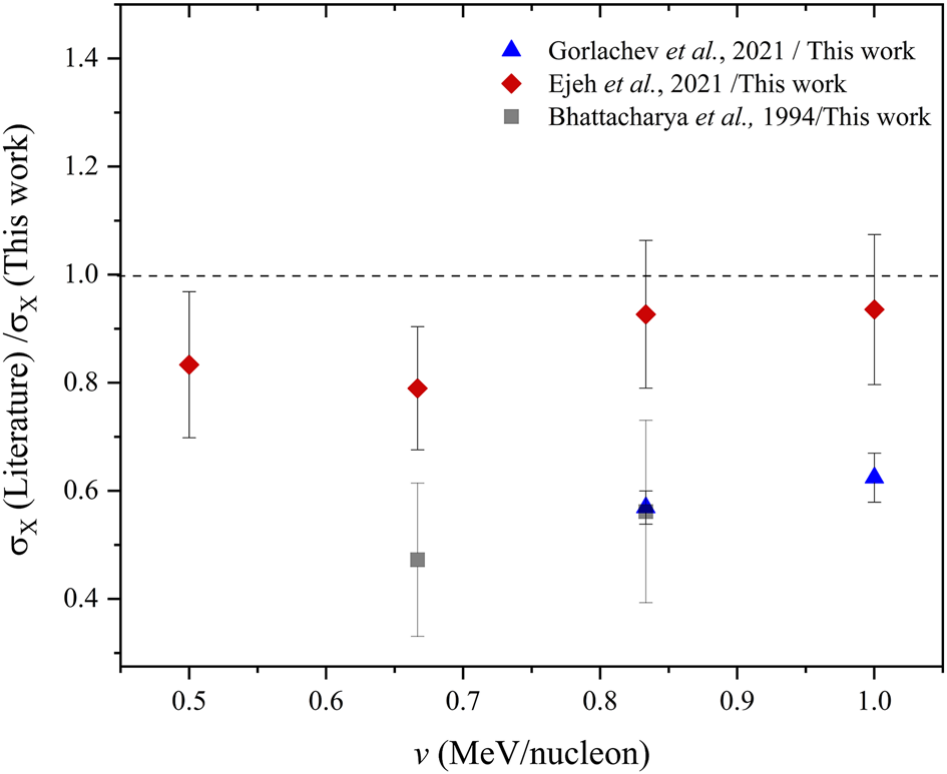
Experimental XPCS ratios due to C in Bi.

The measured cross sections were compared to published datasets available in literature. As shown in Fig. 3, ratios of experimental XPCS comparing our data for C projectiles in Bi showed varying degrees of agreement, at 57–62% with the dataset by Gorlachev et al.^31^, at 47–56% with the dataset by Bhattacharya et al.^32^, and 79–94% for that by Ejeh et al.^21^. The discrepancies of the experimental datasets may be due to experimental uncertainties in the measurement procedure, a challenge commonly seen with XPCS measurements as detailed by Lapicki and Miranda^33^.

Measured HI-XPCS were used to calculate mass and velocity dependent characteristic *R*- ratio datasets (Eq. 1). In the first evaluation, a concurrent analysis of C and F *R*-ratios against ion energy was made. A multi-parameter fit of both datasets was carried out, establishing projectile mass dependent functions of varying offsets but with the same amplitude. It was assumed that all projectiles within the defined projectile mass range exhibited comparable degrees of screening, within negligible degrees of freedom. The function offsets were then used to determine vertical displacement step sizes between the carbon and fluorine *R*-ratios. The *R*-ratio exponential decay function (*also referred to in the text as the reference function*) is shown in Eq. (6).

6 $$f\left(v\right)=A{e}^{(\frac{-v}{t})}+{f(v)}_{z}$$

*where A, v, t and f(v)*_*z*_* are the function amplitude, projectile ion energy per nucleon, the inverse proportionality constant and the function offset (mass characteristic) respectively.*

The difference between the two offset values for the carbon and fluorine *R*-ratio datasets, representing the total displacement ‘D’ about the function axis, is evaluated using Eq. (7).

7 $$D={f(v)}_{{Z}_{i}}-{f(v)}_{{Z}_{0}}$$

**Figure 4:**
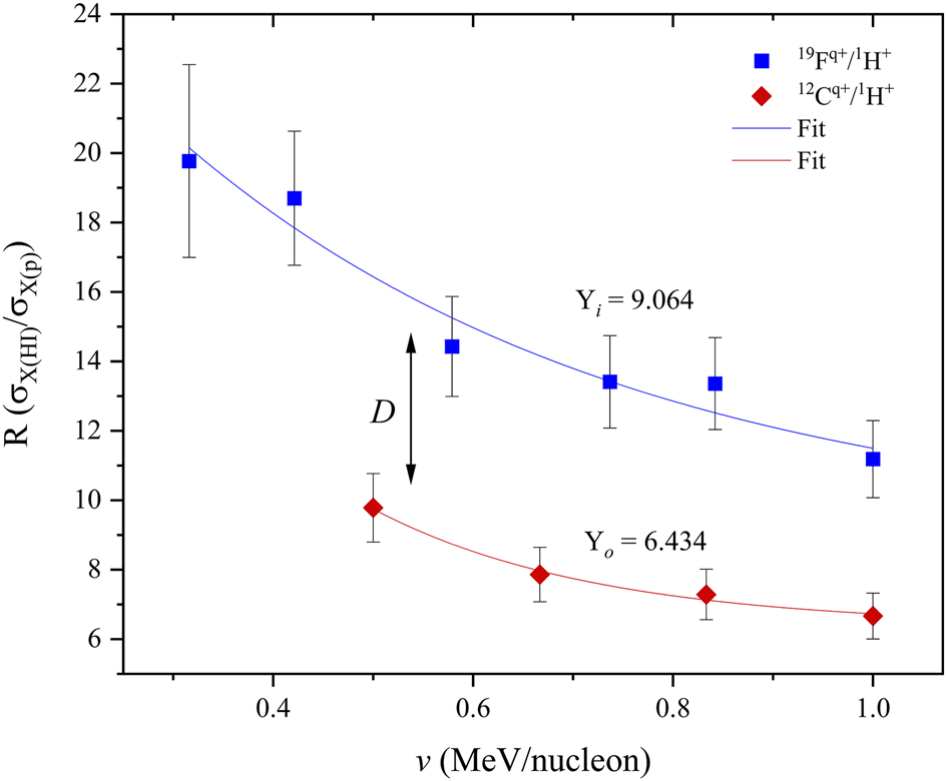
C and F R-ratio function in Bi at (0.3–1.0) MeV/nucleon.

A graphical representation of Eqs. (6) and (7) is shown in Fig. 4; where Y_o_ (or ${f(v)}_{{Z}_{o}}$) and Y_*i*_ (or ${f(v)}_{{Z}_{i}}$) in the *R* plot represents the carbon and fluorine offset values respectively.

As mentioned before, it should be pointed out that the observed deviation from linearity in the R-ratios as the projectile mass increases (see Fig. 1) is mainly attributed to the pronounced MI effects seen with larger projectile ions. This observation thus points to the (mass) characteristic nature of the *R*-ratio trends, implying the need for projectile class groups in the analysis of XPCS ratios for XPCS approximations.

Using the *D* value, the mass dependent displacement step sizes can be calculated using a quotient of *D* and the total number of steps (i.e. atomic mass units) from C to F. The number of steps were determined using the mass difference between the projectiles, where (in the general case) the offset becomes negative if the mass of the projectile whose R-ratio function to be calculated is less than that of the reference ion, in this case C. The mass difference is simply:

8 $$\Delta m={m}_{i}-{m}_{0}$$

*where m*_*i*_* and m*_*0*_* are the masses of the heavier and the lighter/baseline projectile ions (in this case fluorine and carbon) respectively.*

Using the total displacement ‘*D*’ and the total number of steps ‘Δm’, the mass displacement step size is described by the expression: $K=\frac{D}{\Delta m}$.

The *R*-ratio offset in Eq. (5), can now be written as:

9 $${f(v)}_{z}={f(v)}_{{Z}_{0}}+K({m}_{i}-{m}_{0})$$

Inclusive of this mass dependent function offset, the *R*-ratio function is finally defined by Eq. (10) as:

10 $$f\left(v\right)=A{e}^{(\frac{-v}{t})}+\left[{f(v)}_{{Z}_{0}}+K({m}_{i}-{m}_{o})\right]$$

## Results and discussion

### Semi-empirical approximations

Experiment to ECPSSR proton XPCS ratios (i.e. *R*) were determined for C and F ion beams in Bi within the (0.3–1.0) MeV/nucleon ion velocity range. Theoretical proton X-ray production cross sections were translated from ionization cross sections calculated using the ERCS08 code^34^, using *Campbell* recommended fluorescence yields ${\omega }_{i}$ (*i* = 1, 2, 3), relative transition ${\Gamma }_{{L}_{p}}$ (p = *l*, α, β, γ) and total subshell ${\Gamma }_{{L}_{i}}$ (*i* = 1, 2, 3) emission rates and Coster-Kronig transition probabilities *f*_*i*_ (*i* = 12, 13, 23) from the ANSTO database^35^. The translation was carried out using the expressions in (11).

11 $$\begin{aligned} {\sigma }_{X}^{{L}_{l}}&=\left({\sigma }_{L1}({f}_{13}+{f}_{12}{f}_{23})+{\sigma }_{L2}{f}_{23}+{\sigma }_{L3}\right) \cdot {\omega }_{3}\frac{{\Gamma }_{{L}_{l}}}{{\Gamma }_{{L}_{3}}} \\ {\sigma }_{X}^{{L}_{\alpha }} & =\left({\sigma }_{L1}({f}_{13}+{f}_{12}{f}_{23})+{\sigma }_{L2}{f}_{23}+{\sigma }_{L3}\right) \cdot {\omega }_{3}\frac{{\Gamma }_{{L}_{\alpha }}}{{\Gamma }_{{L}_{3}}} \\ {\sigma }_{X}^{{L}_{\beta }} & ={\sigma }_{L1}{\omega }_{1}\frac{{\Gamma }_{{L}_{\beta }}}{{\Gamma }_{{L}_{1}}}+\left({\sigma }_{L1}{f}_{12}+{\sigma }_{L2}\right) \cdot {\omega }_{2}\frac{{\Gamma }_{{L}_{\beta }}}{{\Gamma }_{{L}_{2}}}+\left[{\sigma }_{L1}\left({f}_{13}+{f}_{12}{f}_{23}\right)+{\sigma }_{L2}{f}_{23}+{\sigma }_{L3}\right] \cdot {\omega }_{3}\frac{{\Gamma }_{{L}_{\beta }}}{{\Gamma }_{{L}_{3}}} \\ {\sigma }_{X}^{{L}_{\gamma }} & ={\sigma }_{L1}{\omega }_{1}\frac{{\Gamma }_{{L}_{\gamma }}}{{\Gamma }_{{L}_{1}}}+({\sigma }_{L1}{f}_{12}+{\sigma }_{L2}) \cdot {\omega }_{2}\frac{{\Gamma }_{{L}_{\gamma }}}{{\Gamma }_{{L}_{2}}} \end{aligned}$$

Uncertainties for ECPSSR calculations were attributed to the use of experimental atomic parameters from the recommended values in the translation of ionization to X-ray production cross sections, which were no more than 10%.

The semi-empirical *R*-ratio function offset for the carbon–fluorine reference function (used to calculate relatively low projectile mass datasets) is defined by Eq. (12).

12 $${f(v)}_{z}=6.434+0.376({m}_{2}-12)$$

where m_*2*_ is the mass of the investigated projectile in Bi.

The *R*-ratio function in the C–F projectile mass range is thus defined by Eq. (13).

13 $$f\left(v\right)=27.84{e}^{\left(\frac{-v}{0.348}\right)}+\left[6.434+0.376({m}_{2}-12)\right]$$

**Figure 5:**
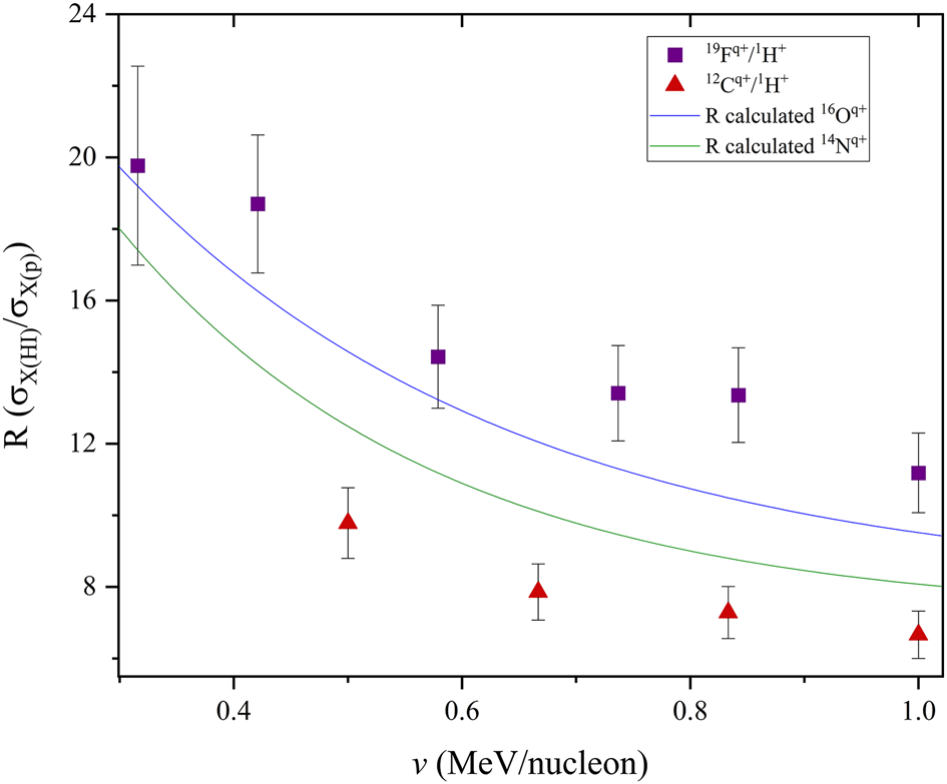
Calculated N and O in Bi at (0.3–1.0) MeV/nucleon.

N and O *R*-ratios in Bi were then calculated using Eq. (13), and are presented in Fig. 5.

What follows is the calculation of HI-XPCS using Eq. (1), for both N and O ions in Bi. Overall uncertainties were determined using calculated experimental uncertainties ranging between 10 and 15% for heavy ions, and no more than 10% for ECPSSR proton cross sections across all ion energies. The mathematical description is given in Eq. (14).

14 $$\Delta U=\sqrt{{\left({U}_{experiment}\right)}^{2}+{\left({U}_{Theory}\right)}^{2}}$$

The results are shown in Table 2.

**Table 2:** Total L-shell N and O XPCS in Bi (in barns) calculated using Eq. (1).

Ion	*v* (MeV/nucleon)	Calculated (*R*)	ECPSSR (DI)
O	0.32	1.24 ± 0.1	0.97
	0.4	4.1 ± 0.5	2.9
	0.5	11.1 ± 1.3	7.53
	0.7	35.4 ± 4.2	27.5
	0.9	72.8 ± 8.7	65.1
	1.0	97.7 ± 12.0	91.4
N	0.32	1.1 ± 0.1	0.93
	0.4	3.6 ± 0.4	2.7
	0.5	9.5 ± 1.1	7.03
	0.7	29.7 ± 3.6	25.2
	0.9	61.3 ± 7.4	59
	1.0	82.9 ± 10.0	82.3

**Figure 6:**
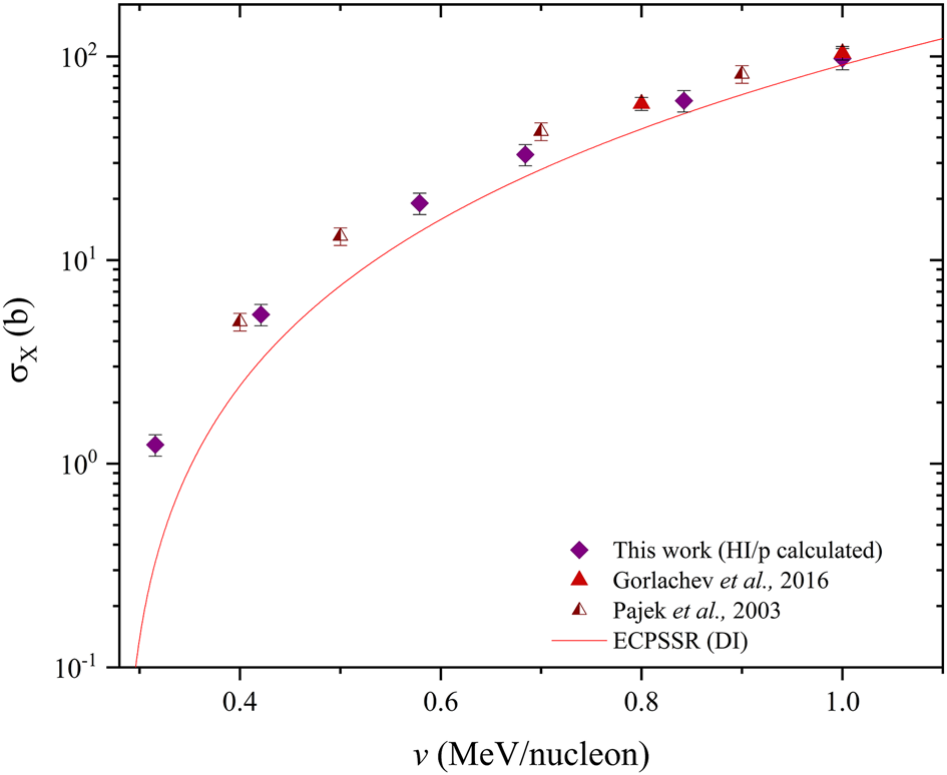
R calculated O induced XPCS in Bi.

The semi-empirical heavy ion induced X-ray production cross sections for O in Bi are shown in Fig. 6, compared to the ECPSSR in direct ionization mode as well as experimental data by Gorlachev et al.^36^, and Pajek et al.^22^.

**Figure 7:**
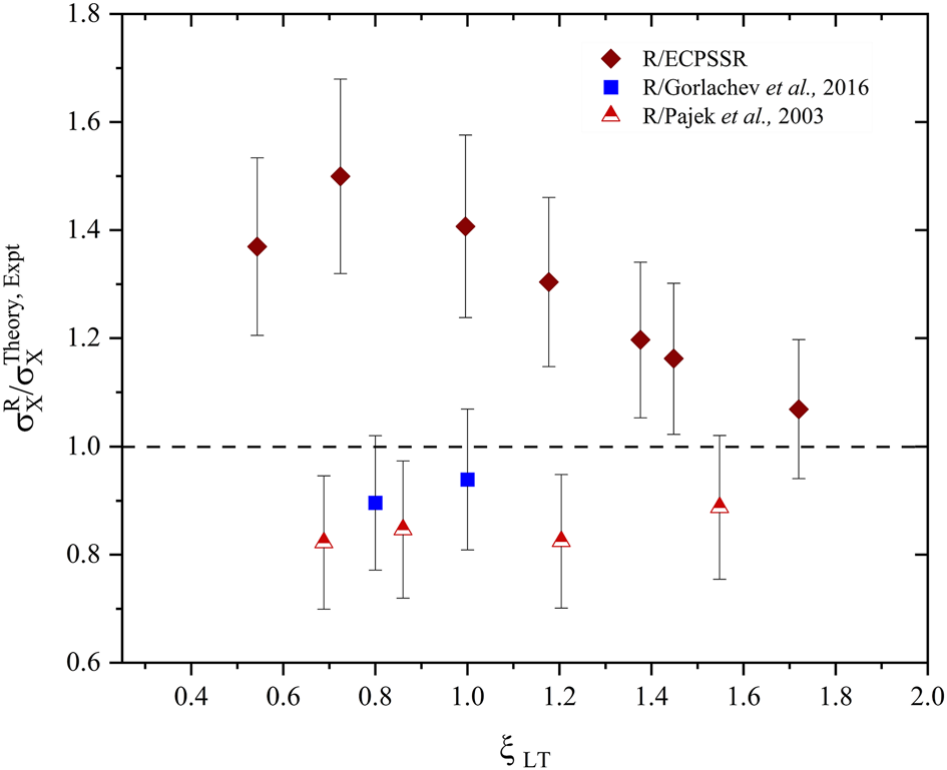
R-ratio vs ECPSSR and experimental XPCS ratios of O ions in Bi.

Figure 6 illustrates a successful account of approximated oxygen XPCS in bismuth. A comparison of estimated R-ratio and ECPSSR theoretical cross sections was carried out to assess relative discrepancies (Fig. 7). As expected, the estimated and ECPSSR cross sections were seen to edge closer to agreement with increasing ion velocity. This, as already mentioned, is due to an increase in projectile ion charge states as the projectile velocity increases. This subsequently reduces significant levels of radiative Electron Capture (EC), particularly due to Multiple Ionisation (MI) effects. For proper analysis, the projectile ion velocity here is translated to the reduced velocity parameter, to compare the projectile and electron velocities (*v*_1_/*v*_2L_) at ion-atom impact. The reduced velocity is described by Eq. (15)^6^.

15 $${\xi }_{{L}_{i}}={2v}_{1}/{\theta }_{{L}_{i}}{v}_{{L}_{i}}$$

The computation of the reduced velocity parameter incorporates the projectile (v_1_) and electron velocities (v_Li_) in the L-shell (*i* = 1, 2, 3). The reduced electron binding energy θ_Li_ is given by ${\theta }_{{L}_{i}}={n}^{2}{U}_{2L}/{Z}_{2{L}_{i}}^{2}\mathcal{R}$ comprised of the terms: *n*—principal quantum number, U_2L_—experimental target electron binding energy, the screened nuclear charge—Z_2Li_^2^ and the Rydberg constant *R*. The screened nuclear charge is described by: ${Z}_{{2L}_{i}}^{2}={Z}_{2}-\mathrm{4,15}$. Since the total L-shell was evaluated, a mean of the reduced velocities for the subshells (i = 1, 2, 3) was used instead.

XPCS ratios show an agreement ranging between 80–95% for the R-ratio vs experimental data across the (0.2–1.0) MeV/nucleon velocity range. Discrepancies between R-ratio datasets and theoretical cross section data are seen at ~ 40–50% at lower ion velocities, where, on the other hand, agreement with available experimental data is up to ~ 95% at 1.0 MeV/nucleon. Agreement with theory was also seen to increase linearly with increasing velocity, compared to experimental data by Pajek^22^ which agreed at a uniform range of ~ 80–90% across all reported velocities. These ratios shown in Fig. 7 suggest convergence to unity beyond 1.0 MeV/nucleon. However, velocities past 1.0 MeV/nucleon were not investigated in this work as this is beyond the scope of heavy ion PIXE within current accelerator limitations. Further predictions in Bi due to Na and Si were carried out. The calculations are presented in Table 3.

**Table 3:** Semi-empirical total L-shell X-ray production cross sections in Bi (in barns) due to Na and Si.

Ion	v (MeV/nucleon)	R (calculated)	ECPSSR (DI)	ECPSSR (DI + EC)
Si	0.4	2.6 ± 0.4	2.6	3.6
	0.6	11.7 ± 2	15.8	24.2
	0.8	30.1 ± 5	48.1	80.2
	1.0	48.7 ± 7	106	192
Na	0.4	2.3 ± 0.4	3.0	3.4
	0.6	10.5 ± 2	17.1	20.6
	0.8	26.4 ± 4	50.1	62.2
	1.0	42.2 ± 6	107	138

**Figure 8:**
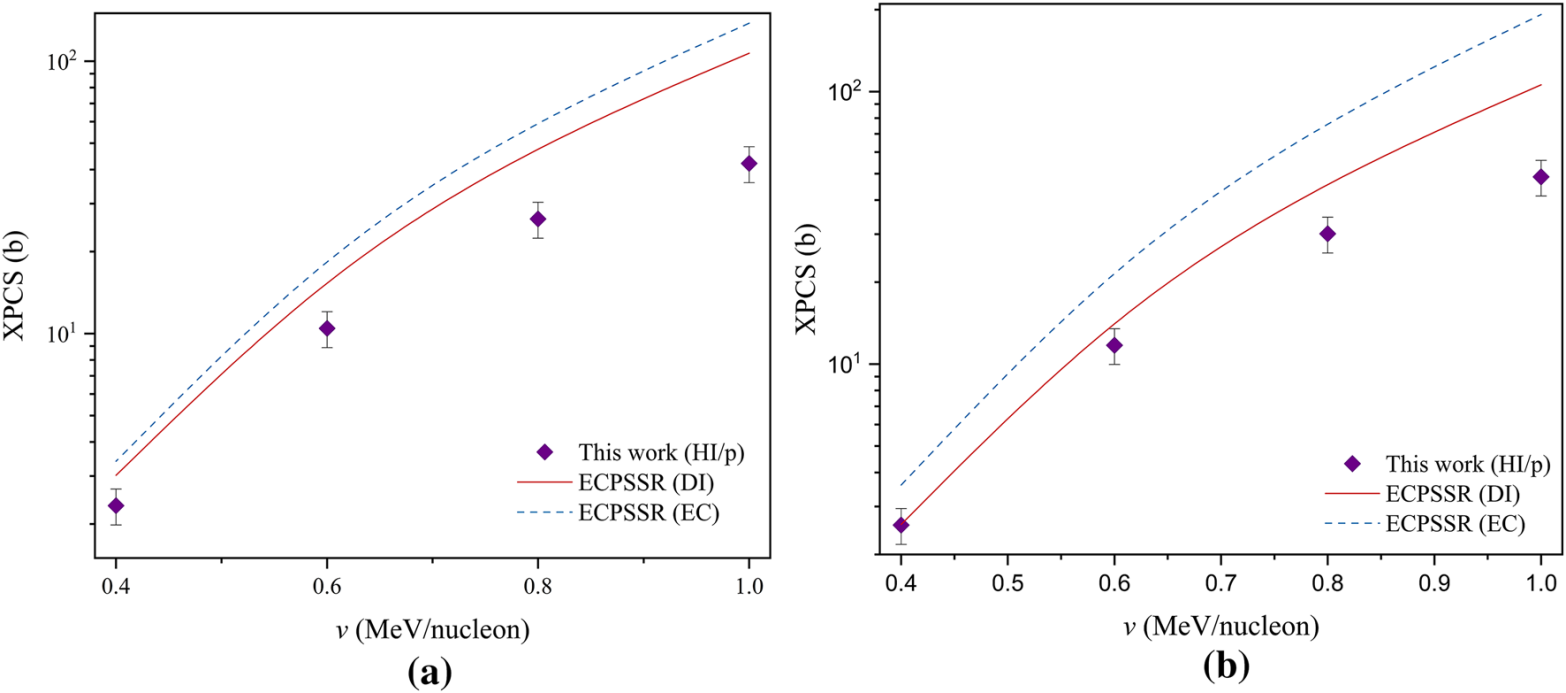
Total L-shell X-ray production cross sections in Bi due to (**a**) Na and (**b**) Si.

The cross sections were compared to predictions by the ECPSSR theory in Direct Ionization (DI) mode as well as the ECPSSR corrected for Electron Capture (EC) effects, shown in Fig. 8.

**Figure 9:**
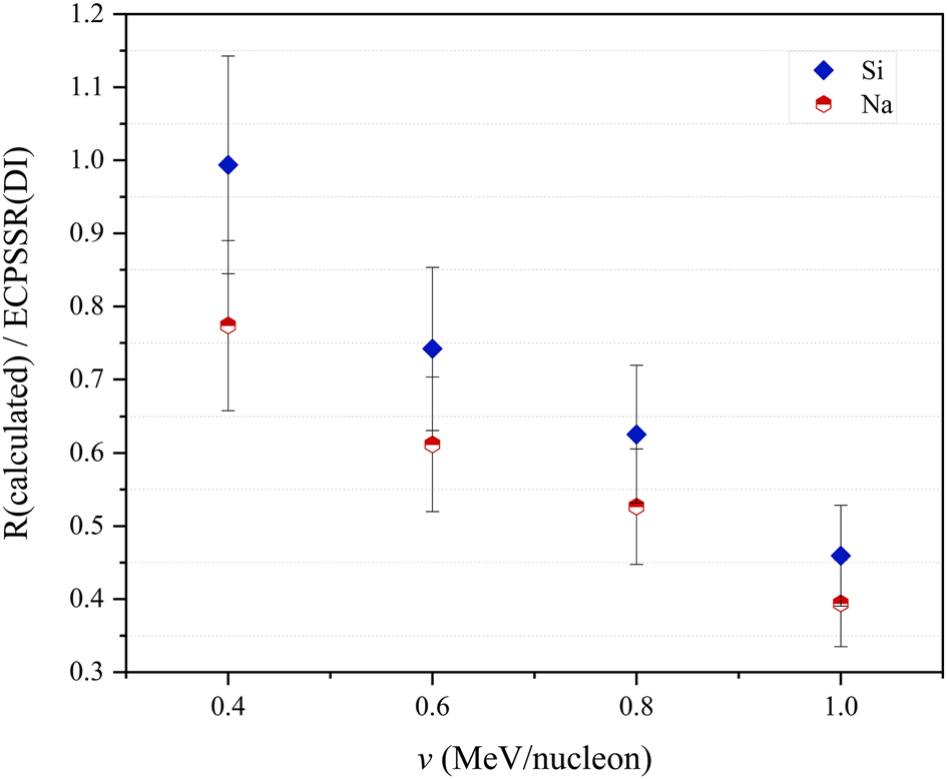
R/ECPSSR XPCS ratios due to Na and Si ionisations.

Unfortunately, previously published experimental data was found to be insufficient to compare with this dataset in order to draw significant conclusions. However, it can be seen that the calculated XPCS converge towards the ECPSSR in both modes with reducing ion velocity. The overall discrepancies between this data and the ECPSSR (DI) ranged at 46–99% for Si and 39–77% for Na, with the highest agreements seen at lower energies. The correlation of semi-empirical vs theoretical data can be seen through XPCS ratios, as shown in Fig. 9.

**Figure 10:**
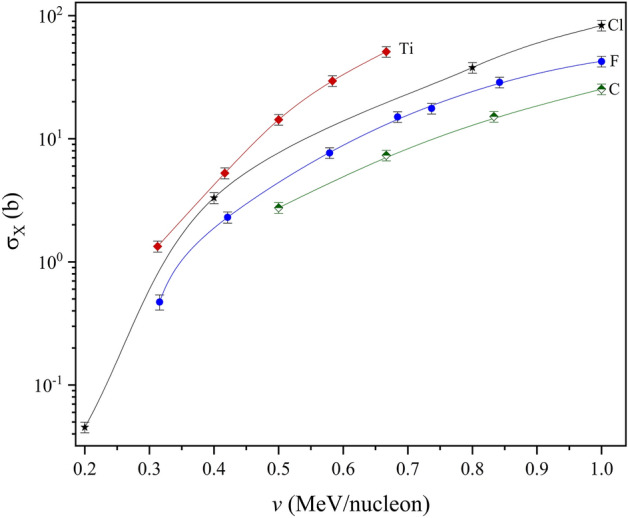
C–Ti experimental XPCS in Bi. Experimental data represented by line + symbols.

Larger discrepancies for Na cross sections compared to Si were observed, with the difference between both datasets ranging between 9 and 13%, pointing to a slightly non-uniform statistical distribution for the same *R* function with increasing ion energy. This implies the reducing validity of the reference semi-empirical function due to carbon and fluorine for Na and Si ionisations, as the function constituent masses are lower than Na. While experimental data is needed to validate this hypothesis, the discrepancies are low and therefore infer a degree of acceptability in the low energy range. This is more so the case for Na, which is only four atomic mass units above F. Considering that both Na and Si resemble quasi-binary collisions in a large target system like Bi (especially for L-subshell ionisations), the ECPSSR may therefore be assumed at higher energies where the *R* model fails. Although F–Cl *R*-ratios would be ideal for calculating Na and Si XPCS, such an approach is unfortunately limited by enhanced XPCS function gradients due to MI effects, as seen in the C–Ti projectile mass range (Fig. 10).

Unlike in the case of proton beams, as shown by Lapicki and Miranda^33^, where a universal empirical fit of XPCS comprised of several ion-atom combinations could be used for the prediction of empirical cross section data; the distortion of HI XPCS trends due to Multiple Ionization effects imply non-universality for heavy ion-atom collisions. It therefore becomes clear that R-ratio predictions cannot be based on a single reference *R* function (e.g. carbon–fluorine). While the exponential cross section gradients due to fairly light heavy ions (such as carbon or fluorine) are comparable, it is clear that Multiple Ionisation effects significantly increase XPCS gradients with increasing projectile mass. This is shown in Fig. 10, and was also confirmed by the comparative *R* XPCS ratios between carbon and chlorine in Fig. 1. This means that *R* reference functions ought to be determined within specific projectile classifications (by order of mass) according to the gradient shifts of the *R* functions. The isolation of *R* reference functions thus ought to be on the basis of projectile-target collision symmetries, with respect to the same projectile and different target masses.

## Conclusion

Carbon and fluorine induced X-ray production cross sections were measured in Bi over an ion velocity range of (0.2–1.0) MeV/nucleon. This was followed by successful parameterization of both C-proton and F-proton (i.e. *R*-ratio) X-ray production cross section ratios which were used to carry out approximations of cross sections for nitrogen, oxygen, sodium and silicon in the same target. Predictions were validated by published experimental data, showing agreement above 80% even at low ion velocities for oxygen ions. Cl and Ti induced XPCS in Bi were also measured; unfortunately, experimental limitations in the ion energies obtained for Ti interactions impeded accurate semi-empirical calculations for ions within the Cl–Ti mass range. Additional experimental data is therefore needed, particularly in the low energy range for titanium induced ionisations in Bi. To this end, the validity of this method requires additional work to establish limitations where different ion-target collision symmetries are concerned.

Future work should thus aim at consolidating existing Cl and Ti induced XPCS in Bi in order to test heavier *R* reference functions for low velocity predictions. This should be followed by measurements of other selected heavy ions, as it has been shown that *R*-ratios cannot be based on a single reference function. Therefore, projectile-target combinations with ions such as Cu should be selected within the framework of Heavy Ion Beam Analysis techniques for potential synergistic applications. Finally, experimental XPCS measurements should be carried out systematically to validate *R* calculations covering a wider projectile-target range.

## Data Availability

The data generated by this study is included in this article.
